# *In Ovo* Administration of Silver Nanoparticles and/or Amino Acids Influence Metabolism and Immune Gene Expression in Chicken Embryos

**DOI:** 10.3390/ijms16059484

**Published:** 2015-04-27

**Authors:** Subrat K. Bhanja, Anna Hotowy, Manish Mehra, Ewa Sawosz, Lane Pineda, Krishna Prasad Vadalasetty, Natalia Kurantowicz, André Chwalibog

**Affiliations:** 1 Central Avian Research Institute, Indian Council of Agricultural Research, Izatnagar UP-243122, India; E-Mails: subratcari@gmail.com (S.K.B.); dr.manishmehra@gmail.com (M.M.); 2 Department of Veterinary Clinical and Animal Sciences, University of Copenhagen, Frederiksberg 1870, Denmark; E-Mails: anna_hotowy@sggw.pl (A.H.); lanepineda@hotmail.com (L.P.); krish@sund.ku.dk (K.P.V.); 3 Department of Animal Nutrition and Biotechnology, Warsaw University of Life Sciences, Warsaw 02-786, Poland; E-Mails: ewa_sawosz@sggw.pl (E.S.); natalia.kurantowicz@gmail.com (N.K.)

**Keywords:** chicken embryo, silver nanoparticles, cysteine, threonine, metabolism, immune gene expression

## Abstract

Due to their physicochemical and biological properties, silver nanoparticles (NanoAg) have a wide range of applications. In the present study, their roles as a carrier of nutrients and an immunomodulator were tested in chicken embryos. Cysteine (Cys)+NanoAg injected embryos had smaller livers but heavier breasts on the 19th day of embryogenesis. Cys injected embryos had lower oxygen consumption compared to threonine (Thr) or NanoAg injected embryos. The energy expenditure in Thr+NanoAg, or NanoAg injected embryos was higher than Cys or Cys+NanoAg but was not different from uninjected control embryos. Relative expression of the hepatic insulin-like growth factor-I (*IGF-*I) gene was higher in Cys or NanoAg injected embryos after lipopolysaccharide (LPS) induction. The gene expression of hepatic tumour necrosis factor-alpha (*TNF-α*) and interleukin-6 (*IL-6*) did not differ among amino acids, NanoAg and uninjected controls in the non-LPS groups, but increased by many folds in the LPS treated NanoAg, Cys and Cys+NanoAg groups. In LPS treated spleens, *TNF-α* expression was also up-regulated by NanoAg, amino acids and their combinations, but interleukin-10 (*IL-10*) expression was down-regulated in Thr, Cys or Thr+NanoAg injected embryos. Toll like receptor-2 (*TLR2*) expression did not differ in NanoAg or amino acids injected embryos; however, toll like receptor-4 (*TLR4*) expression was higher in all treated embryos, except for Cys+NanoAg, than in uninjected control embryos. We concluded that NanoAg either alone or in combination with amino acids did not affect embryonic growth but improved immunocompetence, indicating that NanoAg and amino acid complexes can act as potential agents for the enhancement of innate and adaptive immunity in chicken.

## 1. Introduction

Embryonic life plays an important role in imparting good health to the newly hatched chick. In the early post-hatch period, chicks make the metabolic and physiological transition from egg nutrition (*i.e.*, yolk) to exogenous feed. Approximately 2%–5% of hatchlings do not survive the critical post-hatch period because of limited body reserves. Egg yolk and albumen contain substantial amounts of protein for the developing embryo, but a large fraction of these proteins consist of antibodies, produced by the hen during egg laying [1]. These antibodies provide passive immunity until the neonate can mount an effective immune response [2]. In the modern poultry scenario, hatchlings have to spend a quite long time (24–36 h) without feed and water. This results in mobilisation of residual protein as an energy source for the survival of chicks. Efforts have been made to supplement eggs with critical nutrients (*in ovo* feeding/supplementation) for better post-hatch growth [3], gastro-intestinal tract development [4,5], and immunity [6]; this also reduces the dependency of the embryo on the nutritional status of the laying birds.

Silver nanoparticles (NanoAg) have different physicochemical characteristics compared to their larger counterparts because of their very high surface to volume ratio, physical activity and chemical stability [7,8]. The anti-microbial properties of NanoAg have been exploited in the area of water purification systems and the cosmetic industry. The use of NanoAg in the treatment of deadly diseases like avian influenza [9] supports further research on biological systems. It has been indicated that NanoAg may penetrate tissues and cells and lokalize inside the cell [10,11]. Recent studies have shown that *in ovo* supplementation of NanoAg [12,13], either alone or in combination with glutamine [14], improved the growth and immunity status of embryos and chicks. Furthermore, NanoAg congungajted with hydroxyproline enhanced the development of blood vessels [15]. Zielinska *et al.* [16] reported that gold nanoparticles act as carries of nutrients into muscle cells and affect muscle development. *In ovo* administration of NanoAg nanoparticles up-regulates the expression of fibroblast growth factor (*FGF2*) and vascular endothelial growth factor (*VEGF*) [17] which are needed for satellite cell proliferation, differentiation, vasculogenesis and angiogenesis in tissues. In earlier studies, it was also demonstrated that the *in ovo* supplementation of amino acids not only improves embryonic growth, but also the post-hatch performance and immunity in chickens [3,18,19,20].

The immune system is a dynamic network of cells, tissues, and organs that work in coordination to defend the body against attacks by foreign invaders and protects against diseases by identifying self and non-self cells and tissues [21]. Intercellular communication is mediated by a category of signalling molecules called cytokines. These are small secreted proteins, which are critical to the development and function of both the innate and adaptive immune response. They act by binding to specific membrane receptors, which induce intracellular signalling cascades to alter cell functions. The recognition of pathogens leads to the activation of T helper cells (Th). This process involves adaptive events that occur on the surface of antigen presenting cells (APC). APCs collaborate with the innate response via cytokine communication, which steers the differentiation of Th cells into Th1 or Th2 subsets. These T cells activate cytotoxic T cells and macrophages. Individual Toll-like receptors (TLRs) recognise microbial components that are conserved among pathogens, and such recognition initiates the necessary inflammatory immune responses and induces subsequent activation of adaptive immunity [22]. TLRs 1, 2, 4, 5 and 6 are mainly located on the cell surface and primarily recognise bacterial components, while TLRs 3, 7, 8 and 9 are mostly found in endocytic compartments and mainly recognise viral products [23]. NanoAg can interact with the immune system and carry out different functions like binding and reacting with cells or proteins, thereby modulating immune responses [24].

We hypothesised that the antimicrobial property of NanoAg can also affect immune development in the chicken embryo. Nanoparticles can bypass conventional physiological pathways of nutrient distribution and can be transported across cell membranes and therefore may act as carriers of nutrients. Until now, very few studies have been conducted to assess the role of NanoAg in immunity. The objective of the present study was to demonstrate how NanoAg alone or in association with amino acids (AA) can act as an inducer of innate or adaptive immunity in chicken embryos. We investigated the effect of NanoAg and the amino acids threonine (Thr) and cysteine (Cys), either separately or in combination, on the metabolic rate (oxygen consumption and energy expenditure), growth and immune response at the molecular level through gene expression during embryogenesis.

## 2. Results

### 2.1. Embryo and Organ Development

At the 16th and 19th day of embryogenesis (ED), significantly (*p* < 0.05) lower moisture loss was observed in NanoAg or placebo injected eggs than the uninjected control group, but there was no difference between AA and AA+NanoAg injected eggs. Overall, NanoAg injected eggs lost less moisture than the non-NanoAg group (Table 1).

**Table 1:** Moisture loss and embryo weight in eggs injected with nanoparticles of silver (NanoAg), threonine (Thr), cysteine (Cys) and their combinations. Measured at 16 and 19 day of embryogenesis (ED).

Treatments	Moisture Loss (%)		Embryo Weight (%)	
	16 ED	19 ED	16 ED	19 ED
One-Way ANOVA *				
NanoAg	7.38 ^ab^	8.52 ^a^	22.24 ^c^	41.81 ^b^
Thr	7.79 ^abc^	10.17 ^cd^	20.13 ^bc^	41.88 ^b^
Cys	8.39 ^bc^	10.00 ^bcd^	15.56 ^a^	38.16 ^ab^
Thr+NanoAg	7.87 ^abc^	8.76 ^abc^	19.75 ^b^	41.60 ^b^
Cys+NanoAg	7.83 ^abc^	10.08 ^bcd^	14.94 ^a^	36.88 ^a^
Placebo	6.99 ^a^	8.64 ^ab^	20.81 ^bc^	41.48 ^b^
Uninjected control	9.08 ^c^	10.53 ^d^	20.64 ^bc^	41.81 ^b^
SEM	0.18	0.19	0.52	0.58
*p*-Value	*p* < 0.05	*p* < 0.01	*p* < 0.01	*p* < 0.05
**Two-Way ANOVA**				
NanoAg	7.38	8.52 ^x^	22.24 ^z^	41.81 ^y^
Thr	7.83	9.23 ^xy^	19.94 ^y^	41.69 ^y^
Cys	8.02	10.05 ^y^	15.14 ^x^	37.30 ^x^
*p*-Value	NS	*p* < 0.01	*p* < 0.01	*p* < 0.01
non-NanoAg	8.43 ^s^	10.33 ^s^	19.70	41.17
NanoAg	7.68 ^r^	9.00 ^r^	19.48	40.50
*p*-Value	*p* < 0.05	*p* < 0.01	ns	ns

* Analysis of variance; Values analysed by one-way ANOVA are the mean of six observations; ^a,b,c,d^ or ^x,y,z^ or ^r,s^ Means bearing different superscripts in a row differ significantly (*p* < 0.05); ns = non-significant (*p* > 0.05).

Cys or Cys+NanoAg injected eggs had significantly lower embryo weight at the 16th and 19th ED (*p* < 0.01), but no difference was observed between NanoAg, Thr or their combination. At the 19th ED, Cys or Cys+NanoAg injected embryos had larger hearts (*p* < 0.05) than the NanoAg injected embryos, but did not differ from the Thr or Thr+NanoAg groups. However, the liver size was smaller (*p* < 0.01) in Cys+NanoAg injected embryos. Thr+NanoAg and Cys+NanoAg injected embryos had a heavier breast weight (*p* < 0.01) than NanoAg, placebo or uninjected controls. The relative spleen size did not differ between NanoAg and AA injected embryos. Irrespective of AA injection, NanoAg injected embryos had a lower liver weight but similar heart, spleen and breast weights than the non-NanoAg injected eggs (Table 2).

**Table 2:** Mean internal organ weights of embryo at 19 day of embryogenesis in eggs injected with nanoparticles of silver (NanoAg), threonine (Thr), cysteine (Cys) and their combinations.

Treatments	Heart	Liver	Breast	Spleen
One-Way ANOVA *				
NanoAg	0.72 ^a^	2.18 ^bc^	3.52 ^a^	0.061
Thr	0.76 ^ab^	2.33 ^cd^	3.72 ^ab^	0.057
Cys	0.82 ^b^	2.19 ^bc^	3.86 ^ab^	0.053
Thr+NanoAg	0.79 ^ab^	2.13 ^b^	4.04 ^bc^	0.058
Cys+NanoAg	0.83 ^b^	1.96 ^a^	4.39 ^c^	0.054
Placebo	0.75 ^ab^	2.44 ^d^	3.35 ^a^	0.060
Uninjected control	0.79 ^ab^	2.22 ^bc^	3.52 ^a^	0.061
SEM	0.01	0.03	0.08	0.002
*p*-Value	*p* < 0.05	*p* < 0.01	*p* < 0.01	ns
**Two-Way ANOVA**				
NanoAg	0.72 ^x^	2.18	3.52 ^x^	0.061
Thr	0.78 ^xy^	2.23	3.88 ^xy^	0.058
Cys	0.83 ^y^	2.05	4.18 ^y^	0.053
*p*-Value	*p* < 0.01	ns	*p* < 0.05	ns
non-NanoAg	0.79	2.25 ^s^	3.68	0.058
NanoAg	0.78	2.09 ^r^	3.98	0.057
*p*-Value	ns	*p* < 0.05	ns	ns

* Analysis of variance; Values analysed by one-way ANOVA are the mean of six observations; ^a,b,c,d^ or ^x,y,z^ or ^r,s^ Means bearing different superscripts in a row differ significantly (*p* < 0.05); ns = non-significant (*p* > 0.05).

### 2.2. Gaseous Exchange and Energy Expenditure

Embryo O_2_ consumption (mL/h) on the 10th, 16th and 19th ED was similar in NanoAg, uninjected control and Thr+NanoAg injected eggs, and was significantly lower in Cys or Thr or Cys+NanoAg injected embryos. Embryo energy expenditure (EE) on the 10th ED was higher in NanoAg injected embryos than in Thr or Cys injected embryos, but did not differ from the uninjected control group. Beyond the 13th ED, EE was lower (*p* < 0.01) in Cys injected embryos, but the addition of NanoAg did not affect EE. The Thr+NanoAg injected embryos had a similar EE level to that of the NanoAg and uninjected control groups. Irrespective of the nutrients injected, the NanoAg group had significantly greater O_2_ consumption and EE than their non-NanoAg counterparts (Table 3).

### 2.3. Growth Gene Expression

**Figure 1:**
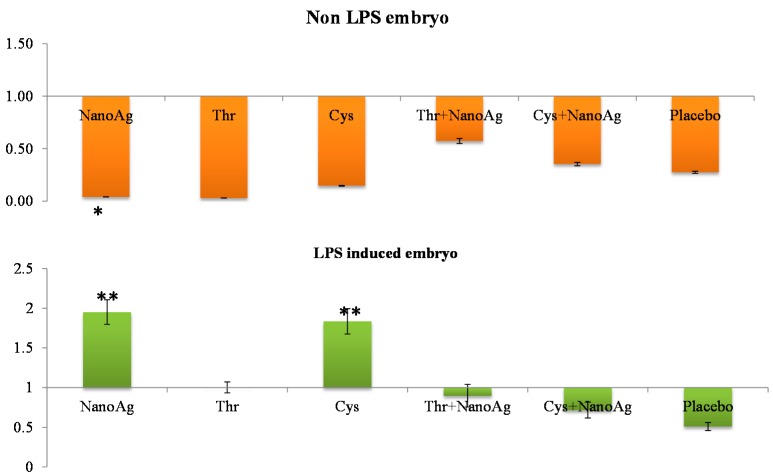
Relative fold expression level of insulin-like growth factor-I (*IGF-I*) gene in hepatic tissues of normal (non-LPS) and lipopolysaccharide (LPS) induced *in ovo* NanoAg and/or amino acid injected embryos. Expression of uninjected control embryo is taken as 1.0. * and ** indicates significant expression at the level of 95% (*p* < 0.05) and 99% (*p* < 0.01), respectively.

Relative gene expression of hepatic IGF-1 in 19th ED embryos showed no significant changes (*p* > 0.05) between AA+NanoAg injected eggs compared to the uninjected control, but NanoAg injected embryos had significantly lower (*p* < 0.05) expression (down-regulation) compared to the uninjected control. Upon LPS treatment, *IGF-I* gene expression was up-regulated (*p* < 0.05) in the NanoAg or Cys injected groups but not in the other treatments (Figure 1).

**Table 3:** Oxygen consumption and energy expenditure from chicken embryo during 10–19 days of embryogenesis (ED) in eggs injected with nanoparticles of silver (NanoAg), threonine (Thr), cysteine (Cys) and their combinations.

Treatments	Oxygen Consumption (mL/h)				Energy Expenditure (Joules/h)			
	10 ED	13 ED	16 ED	19 ED	10 ED	13 ED	16 ED	19 ED
One-Way ANOVA *								
NanoAg	3.46 ^c^	8.79	25.74 ^d^	33.67 ^c^	65.8 ^b^	167.5 ^ab^	498.7 ^d^	657.8 ^c^
Thr	2.91 ^ab^	9.01	23.07 ^b^	30.89 ^b^	55.9 ^a^	171.8 ^ab^	444.2 ^b^	599.6 ^b^
Cys	2.81 ^a^	7.76	21.34 ^a^	24.42 ^a^	55.1 ^a^	147.8 ^a^	410.2 ^a^	468.8 ^a^
Thr+NanoAg	3.13 ^abc^	9.54	24.57 ^c^	33.10 ^c^	61.0 ^ab^	183.2 ^bc^	477.6 ^c^	645.7 ^c^
Cys+NanoAg	2.92 ^ab^	7.57	22.78 ^b^	29.97 ^b^	57.1 ^a^	144.4 ^a^	442.3 ^b^	586.5 ^b^
Uninjected control	3.26 ^bc^	8.72	26.35 ^d^	32.60 ^c^	62.2 ^ab^	166.6 ^ab^	512.9 ^d^	644.3 ^c^
SEM	0.053	0.22	0.29	0.67	0.96	4.31	5.91	13.84
*p*-Value	*p* < 0.01	ns	*p* < 0.01	*p* < 0.01	*p* < 0.01	ns	*p* < 0.01	*p* < 0.01
**Two-Way ANOVA**								
NanoAg	3.46 ^z^	8.79 ^xy^	25.74 ^z^	33.67 ^y^	65.8 ^y^	167.5 ^xy^	498.7 ^z^	657.8 ^y^
Thr	3.02 ^y^	9.27 ^y^	23.82 ^y^	31.99 ^y^	58.5 ^x^	177.5 ^y^	460.9 ^y^	622.7 ^y^
Cys	2.86 ^x^	7.66 ^x^	22.06 ^x^	27.19 ^x^	56.1 ^x^	146.1 ^x^	426.2 ^x^	527.6 ^x^
*p*-Value	*p* < 0.01	*p* < 0.05	*p* < 0.01	*p* < 0.01	*p* < 0.01	*p* < 0.05	*p* < 0.01	*p* < 0.01
non-NanoAg	2.99 ^r^	8.49	23.59 ^r^	29.30 ^r^	57.8 ^r^	162.0	455.8 ^r^	570.9 ^r^
NanoAg	3.17 ^s^	8.64	24.37 ^s^	32.25 ^s^	61.3 ^s^	165.0	472.9 ^s^	630.0 ^s^
*p*-Value	*p* < 0.05	ns	*p* < 0.01	*p* < 0.01	*p* < 0.05	ns	*p* < 0.01	*p* < 0.01

* Analysis of variance; Values analysed by one-way ANOVA are the mean of six observations; ^a,b,c,d^ or ^x,y,z^ or ^r,s^ Means bearing different superscripts in a row differ significantly (*p* < 0.05); ns = non-significant (*p* > 0.05).

### 2.4. Immune Gene Expression

#### 2.4.1. In Hepatic Tissue

**Figure 2:**
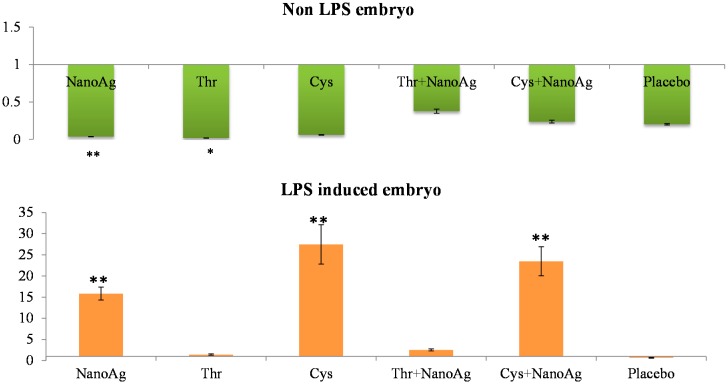
Relative fold expression level of interleukin-6 (*IL-6*) gene in hepatic tissues of normal (non-LPS) and LPS induced *in ovo* NanoAg and/or amino acid injected embryos. Expression of uninjected control embryo is taken as 1.0. * and ** indicates significant expression at the level of 95% (*p* < 0.05) and 99% (*p* < 0.01), respectively.

There was significant down-regulation of hepatic *IL-6* gene expression in AA and NanoAg injected embryos, but not in the AA+NanoAg groups compared to the uninjected control. However, upon treatment with LPS, the relative gene expression of *IL-6* increased in NanoAg (16.7-fold), Cys (29.4-fold) and Cys+NanoAg (25.0-fold) injected embryos but not in Thr or uninjected control embryos (Figure 2).

**Figure 3:**
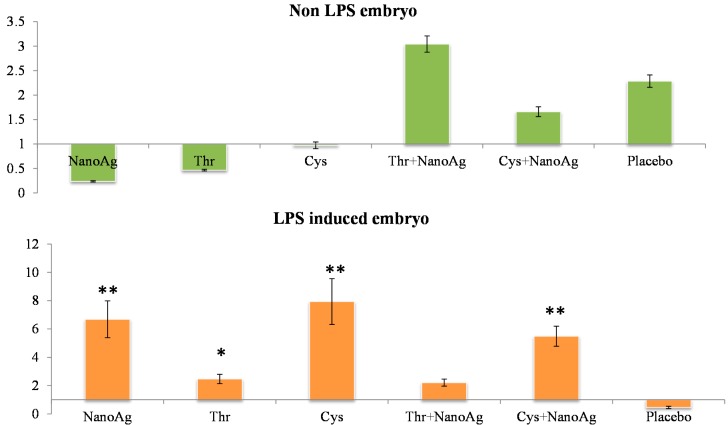
Relative fold expression level of tumour necrosis factor-alpha (*TNF-α*) gene in hepatic tissues of normal (non-LPS) and LPS induced *in ovo* NanoAg and/or amino acid injected embryos. Expression in uninjected control embryo is taken as 1.0. * and ** indicates significant expression at the level of 95% (*p* < 0.05) and 99% (*p* < 0.01), respectively.

**Figure 4:**
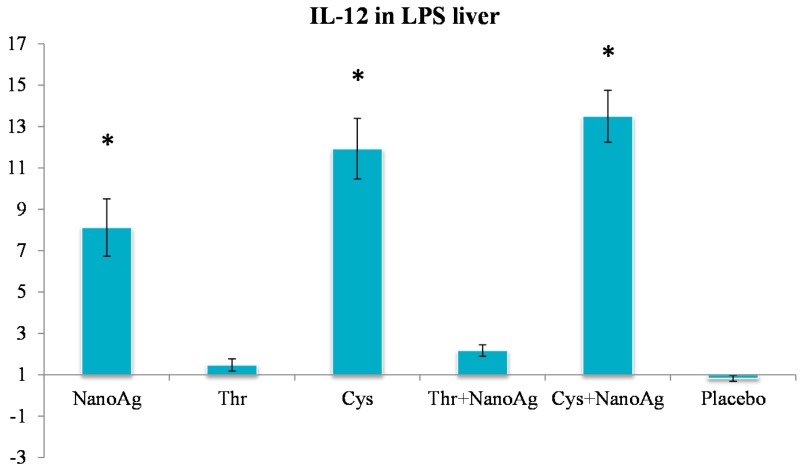
Relative fold expression level of interleukin-12 (*IL-12*) gene in hepatic tissues of LPS induced *in ovo* NanoAg and/or amino acid injected embryos. Expression of uninjected control embryo is taken as 1.0. * indicates significant expression at the level of 95% (*p* < 0.05).

In the non-LPS groups, the expression of *TNF-α* was not different in AA treated, NanoAg treated and uninjected gene control embryos. Following LPS treatment, *TNF-α* gene expression increased in NanoAg, Thr, Cys and Cys+NanoAg embryos but not in Thr+NanoAg embryos compared to uninjected control embryos (Figure 3). Significantly higher expression of interleukin-12 (*IL-12*) was observed in NanoAg, Cys or Cys+NanoAg embryos than in uninjected embryos following LPS treatment (Figure 4).

#### 2.4.2. In Spleen Tissue

**Figure 5:**
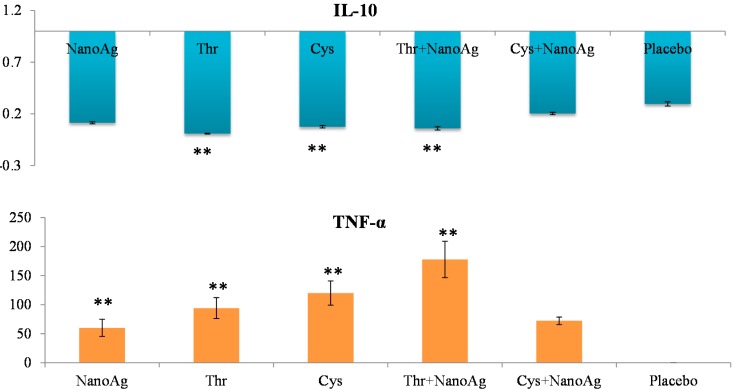
Relative fold expression level of interleukin-10 (*IL-10*) and tumour necrosis factor-alpha (*TNF-α*) gene in spleen tissues of LPS induced *in ovo* NanoAg and/or amino acid injected embryos. Expression of uninjected control embryo is taken as 1.0. ** indicates significant expression at the level of 99% (*p* < 0.01).

Differential gene expression of TNF-α, IL-10, TLR2 and TLR4 was studied in the spleen samples of LPS treated embryos on the 19th ED. There was significant up-regulation of TNF-α in NanoAg, AA or their combinations compared to the uninjected control group. Significant down-regulation of IL-10 was observed in Thr, Cys or Thr+NanoAg injected embryos compared to the uninjected control group. The other treatment groups also showed down-regulation, but this did not reach the level of significance (Figure 5).

**Figure 6:**
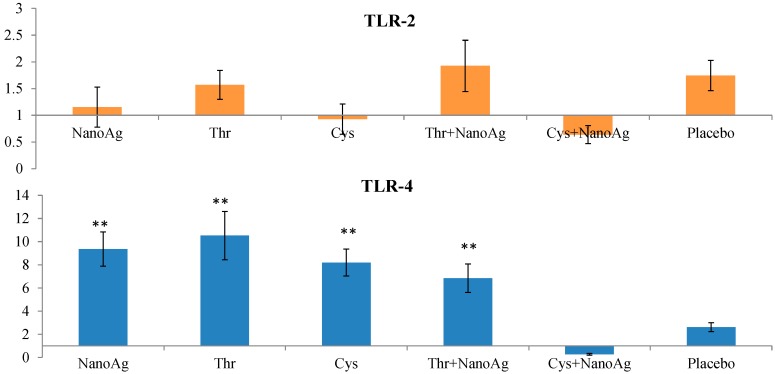
Relative fold expression level of toll like receptor-2 (*TLR-2*) and toll like receptor-4 (*TLR-4*) gene in spleen tissues of LPS induced *in ovo* NanoAg and/or amino acid injected embryos. Expression of uninjected control embryo is taken as 1.0. ** indicates significant expression at the level of 99% (*p* < 0.01).

Relative TLR2 expression did not differ between AA, NanoAg or their combinations and the uninjected control group. However, TLR4 expression was significantly (*p* < 0.05) up-regulated in NanoAg (9.36-fold), Thr (10.53-fold), Cys (8.19-fold) and in Thr+NanoAg (6.85-fold) compared to the placebo and uninjected control groups (Figure 6).

## 3. Discussion

Except for the Cys injected groups, embryo development was similar with NanoAg, Thr and their combination compared to the uninjected control and placebo groups. Earlier studies in broiler and layer chickens suggested that nanoparticles of Ag and Au neither improved nor depressed embryonic growth [25], but affected embryo organ size [13]. In this study, NanoAg injected embryos had a lower liver weight but similar heart, spleen and breast weight compared to the uninjected embryos. It has also been reported that NanoAg, glutamine and a complex of NanoAg and glutamine did not affect the weight of the liver, heart and spleen of embryos, but the muscle percentage increased significantly with NanoAg+glutamine treatment compared to the other groups [14]. Similar findings in quail, pigs, and chickens support the fact that NanoAg does not affect growth [12,26,27,28]. Andi *et al.* [29] showed that silver (especially in the form of nanoparticles) had negative effects on liver weight, but the bursa and spleen weights were higher, in contrast to the investigation by Ahmadi and Kurdestani [30]. *In ovo* injection of amino acids, especially Thr or methionine, resulted in a higher chick weight to egg weight ratio on the day of hatching [19]. A similar trend of higher embryo weight was observed on the 19th ED in Thr and NanoAg injected eggs in the present study. However, the embryo weight decrease in Cys injected eggs could not be explained, but toxicity to cysteic acid (an intermediate of Cys metabolism) may be one of the reasons as higher mortality (data not provided) was recorded in these groups.

In the present study, reduced moisture loss but similar embryo growth was observed in NanoAg injected eggs compared to the uninjected control group. Although the O_2_ consumption and EE of NanoAg injected embryos was significantly increased, their body and muscle weights at hatching were similar to those of the control group, which suggests that the concentration of NanoAg (50 ppm) was adequate to increase the metabolic rate but not enough to affect growth and development. These results confirm previous findings demonstrating significant effects on the metabolic rate in NanoAg treated layer embryos, but no effect on body and organ weight [31]. In the present study, O_2_ consumption and EE from the 10th–19th ED were higher in NanoAg injected eggs than in eggs injected with AA or their combinations with NanoAg. Additionally, moisture loss was also lower this group. The present results indicate that NanoAg could be a potential modifier of the metabolic rate in layer embryos; however, to understand the exact mechanism of action, more research is required.

Relative gene expression of hepatic *IGF-I* at the 19th ED did not differ between AA and NanoAg injected embryos and the uninjected control; however, this increased in NanoAg or Cys injected embryos following LPS treatment. Earlier studies also reported increased *IGF-I* and chicken growth hormone (*cGH*) gene expression at the 20th ED in Thr, arginine, methionine and Cys treated chicks [32]. These authors suggested that *in ovo* feeding might have helped the embryos to achieve higher skeletal muscle growth by reducing protein degradation and or by increasing protein synthesis [33,34]. Generally, *IGF-I and -II* are responsible for the proliferation of pre-adipocytes, chondrocytes, and fibroblasts through amino acid stimulation, glucose uptake, increased DNA synthesis, tissue growth stimulation, and overall embryogenesis regulation [35,36,37]. In the present study, although the embryo weight was higher in Thr and NanoAg injected embryos, this did not translate to *IGF-I* expression. Hotowy *et al.* [17] reported an increase in the mRNA expression of *FGF2* and *VEGFA* genes. The increase in the gene expression of *IGF-I* in LPS treated Cys injected embryos could be due to increased consumption of Cys as its concentration decreased from 151 mg on the 14th day of incubation to 80 mg on the 19th day of incubation [38]. Cys also added to the glutathione and accessory protein produced by the liver during the acute phase response [39]. Cys also plays an important role in antibody production, which is manufactured in line to switch from a closed system towards an external natural environment.

Immune gene expression was studied in the hepatic and spleen tissues on 19-day-old embryos after treatment with LPS. In the present study, we tried to compare the expression of T helper 1 (Th1; cellular immunity) and Th2 (humoral immunity) cytokines and TLRs, which play vital roles in the innate and adaptive immune systems of birds. It is interesting to note that there was significantly higher expression of humoral and cell-mediated interleukins in response to LPS. It has been reported that LPS induces a strong immune response by the release of pro-inflammatory cytokines, resulting in a generalised inflammatory response, tissue damage and even septic shock [40,41]. The balance between Th1 and Th2 cytokine production determines the early preference for the kind of immune response the individual mounts.

Tumour necrosis factor-α (*TNF-α*) is a key cytokine involved in inflammation and immunity [42], and the pro-inflammatory cytokine *IL-6* induces final maturation of B cells into antibody-secreting plasma cells [43], thereby increasing the secretion of immunoglobulins [44]. In the present study, increased expression of *TNF-α* gene in the livers and spleens of LPS challenged *in ovo* injected NanoAg, Cys and Cys+NanoAg embryos suggested improvement in the immunity status of the embryos. These findings also support earlier studies by Sirimongkolkasem [45], who reported that Cys is in greater demand than other amino acids for humoral immunity. Bhol and Schechter [46] demonstrated that NanoAg inhibit allergic contact dermatitis in mice, similar to steroids and tacrolimus. In the non-LPS groups, *IL-6* or *TNF-α* gene expression was not different between AA and AA+NanoAg injected embryos, but significantly increased in Cys and Cys+NanoAg injected embryos following LPS treatment, suggesting their roles as modulators of humoral immunity involving Th2 cytokines. In an earlier study, *in ovo* Met+Cys injected birds showed higher gene expression of *IL-6* and *TNF-α* than uninjected (control) broiler chickens [32].

Greiner *et al.* [47] reported that glucose is an essential fuel for proliferating Th2 cells for the production of antibodies, and thus humoral immunity. Studies in human diabetic individuals revealed that hyperglycaemia acutely increases circulating cytokine concentrations of *IL-6* and *TNF-α* by an oxidative mechanism, and plays a role in immune activation in diabetes [48]. In another study, Humphrey and Rudrappa [49] reported that glucose availability induces metabolic changes in thymocytes that alters their energy status and influences the development of naive T cells in the chicken thymus. Animals administered Ag nanoparticles in their drinking water showed increased proinflammatory cytokine levels (*IL-1β*, *IL-6* and *TNF-α*) in response to LPS challenge regardless of the nanoparticle dose [50].

In the present study, expression *IL-12* was significantly increased after LPS induction in the groups given NanoAg, Cys or their combination. *IL-12* promotes the differentiation of native CD4^+^ T cells into Th1 cells, increases natural killer (NK) cellular cytotoxicity and the proliferation of T cells, and stimulates the production of interferon-γ (*IFN-γ*) and *TNF-α* [51]. *IL-12* also has anti-angiogenic activity, which it imparts by producing a chemokine called inducible protein-10 (IP-10 or CXCL10) [52]. Because of its ability to induce immune responses and its anti-angiogenic activity, there has been interest in testing *IL-12* as a possible anti-cancer drug. As NanoAg or Cys induce *IL-12* expression, their role in anticancer therapy cannot be ruled out.

Expression of *IL-10* in the spleen tissues of LPS treated embryos was significantly down-regulated in the Thr, Cys or Thr+NanoAg injected groups but not in the NanoAg group. *IL-10* is capable of inhibiting the synthesis of pro-inflammatory cytokines such as *IFN-γ*, *IL-2* and *TNF-α* made by macrophages and regulatory T cells [53]. However, it is also stimulatory towards Th2 cells and mast cells and helps with B cell maturation and antibody production. This was evident in the present study where *TNF-α* expression was increased in LPS treated embryos injected with NanoAg, Cys or Thr. *IL-12* also displays a potent ability to suppress the antigen presentation capacity of antigen presenting cells. A study in mice has shown that IL-10 is also produced by mast cells, counteracting the inflammatory effect that these cells have at the site of an allergic reaction [54]. *IL-10* is released by cytotoxic T cells to inhibit the action of NK cells during the immune response to viral infection in humans [55].

Toll-like receptors play an important role in innate immunity. In the present study, the gene expression of *TLR4*, *IL-6* and *TNF-α* was increased in the NanoAg and Cys groups after LPS treatment. The basic role of TLRs in innate immunity is consistent in mammalian and avian species. Antigen presenting cells such as macrophages and dendritic cells express TLRs on their surface. Upon pathogen infection, pathogen associated molecular patterns are signalling by TLRs present on APCs and initiate a signalling cascade that stimulates the innate defence through the induction of reactive oxygen and nitrogen intermediates. They also initiate adaptive immunity by activating APCs for the production of pro-inflammatory cytokines (*IL-1β*, *IL-6*, chemokines, and *IL-8*) and up-regulating co-stimulatory molecules [56]. Cysteine is important to T cells, as it is a precursor of the tripeptide glutathione. For T cell activation and proliferation, the intracellular glutathione content must be increased to sufficient amounts, and without adequate cysteine levels glutathione synthesis is also limited [57]. In recent years, it has been clearly demonstrated that several amino acids play a significant role in regulating a variety of immune responses, including the activation of lymphocytes, NK cells, and macrophages; proliferation of lymphocytes; regulation of intracellular redox states; gene expression; and production of cytokines [58].

## 4. Experimental Section

### 4.1. Experimental Design

Lohmann (egg type) chicken eggs were obtained from a commercial hatchery and randomly distributed into seven groups of 33 eggs each. Eggs were injected into the air sac with either 0.3 mL of NanoAg solution (15 mg NanoAg per egg), 15.75 mg of amino acids threonine (Thr) and 15.75 mg of cysteine (Cys) or their combinations Thr and NanoAg (Thr+NanoAg), NanoAg and Cys (Cys+NanoAg) dissolved in phosphate buffered saline (PBS). For comparison, two controls were used. The group which received PBS (placebo) solution acted as an injected control while the other group did not receive any treatment and was the uninjected control. Injection was carried out prior to incubation into the air sac using a sterile 27 gauge, 20 mm needle. Immediately after injection, the hole was sealed with sterile tape and the eggs were placed in an incubator. The eggs were incubated for 21 days under standard conditions (temperature 37.8 °C, humidity 55%, turned once per hour during the first 18 days, at a temperature of 37 °C and humidity 60% from day 19 onwards).

### 4.2. Nanosolution

Hydrocolloid Ag solutions were obtained from Nano-Tech, Warsaw, Poland and were produced by a non-explosive high voltage method from high purity metal (99.9999%) and high purity demineralized water. The concentration of nanoparticles in the hydrocolloid was 50 ppm AgNano with a particle size ranging from 2–35 nm based on transmission electron microscope evaluations as described by Chwalibog *et al.* [59].

### 4.3. Oxygen Consumption and Energy Expenditure

Eggs were candled at 10th, 13th, 16th and 19th ED and those showing a developing embryo were weighed. Moisture loss was estimated by subtracting the pre-incubated weight from the 16th and 19th ED weight and represented as percentage of pre-incubated weight. Gas exchange (O_2_ consumption and CO_2_ production) was measured according to the procedure described by Chwalibog *et al.* [60] in an open-air circuit respiration unit (Micro-Oxymax calorimeter from Columbus Instruments, Columbus, OH, USA), equipped with four respiration chambers with a volume of 2000 cm^3^ each. The temperature and relative humidity were maintained similar to that of the incubator. Six eggs each from three treatment groups were placed in each chamber and measured for 2 h in the morning at 8:00–10:00 and other three treatment groups (excluding placebo) at 10:00–12:00. The measurement of gas exchange was repeated on the same day in the afternoon at 12:30–14:30 and 14:30–16:30 with another set of six eggs from the respective treatment groups. After each measurement, the eggs were put back into the incubator. All gas exchange and EE measurements were standardised to a 50 g egg mass in order to account for differences in egg weight during each measurement. EE was calculated using the formula: EE (kJ) = 16.18 × O_2_ (mL) + 5.02 × CO_2_ (mL) [61].

### 4.4. Lipopolysaccharide (LPS) Treatment

On the 19th ED, six eggs per treatment were injected with lipopolysaccharide (LPS), 0.2 mg/egg into the amniotic cavity of the embryo and the eggs were returned back to the incubator. Another group of six eggs per treatment was kept as such in the incubator and acted as a control for LPS treatment (non-LPS). After 4 h, the eggs were opened and the embryos were culled by decapitation to collect liver and spleen tissues for gene expression studies.

### 4.5. Embryo Measurement and Tissue Sampling

On the 16th and 19th ED, six eggs from each treatment were taken out from the incubator, weighed and broken to remove the live embryo. The live embryo weight was recorded after drying with tissue paper. These embryos were then killed by cervical dislocation and the heart, liver, spleen and breast muscle were dissected and weighed. Furthermore, 100 mg liver and spleen tissue samples were collected from each embryo for gene expression studies. The experimental procedures followed Danish National Legislation.

### 4.6. Gene Expression at the mRNA Level

The tissues dissected from the liver and spleen were homogenised in TRIzol^®^ Reagent (Life Technologies, Naerum, Denmark), and total RNA was extracted according to the manufacturer’s instructions. The RNA samples were purified using the SV Total RNA Isolation System (Promega Corporation, Madison, WI, USA) and quantified using a NanoDrop ND 1000 spectrophotometer (Thermo Fisher Scientific, Waltham, MA, USA). Using reverse transcriptase with oligo(dT) (Promega) and random primers (TAG Copenhagen A/S, Copenhagen, Denmark), 2 µg of total RNA was reverse transcribed, after which real-time PCR was performed with complementary DNA and gene-specific primer pairs (Table 4) mixed with 1X QuantiTect SYBR Green PCR master mix (SYBR Green 1 dye), HotStart Taq DNA polymerase and dNTPs in optimised buffer components (QIAGEN GmBH, Hilden, Germany) in a IQ5 cycler (Biorad, Hercules, CA, USA). The samples were first denatured for 5 min at 95 °C and then amplified using 45 cycles of 30 s at 95 °C for denaturation, 30 s at specific annealing temperature (Table 1), and 30 s at 72 °C (elongation), followed by quantification. A melting curve was applied to verify the specificity of the product. For each complementary DNA, the reaction was performed in triplicate.

**Table 4:** Oligonucleotide sequence of growth- and immune-related gene primers.

Gene ^1^	Sequence (5'→3')	Annealing Temp. (°C)	Product Size (bp)	Accession Number	E * (%)
*IGF-I*	F-GGTGCTGAGCTGGTTGATGC	58	203	JN942578	96
	R-CGTACAGAGCGTGCAGATTTAGGT				
*IL-6*	F-GAAATCCCTCCTCGCCAATCTGA	57	281	JN639847	96
	R-TGAAACGGAACAACACTGCCATCT				
*IL-10*	F-CTGAAGGCGACGATGC	57	179	EF554720	99
	R-TTCCTCCTCCTCATCAGC				
*IL-12*	F-GCCGACTGAGATGTTCCTGG	57	227	JN942590	92
	R-CCTTGCTTTTGTATTTCTTTGTGC				
*TNF-α*	F-AGACCAGATGGGAAGGGAATGAA	55	219	JN942589	91
	R-GAAGAGGCCACCACACGACAG				
*TLR2*	F-GTGGCCATGTCGATCAGCAGAAAC	58	202	JQ280465	99
	R-TCAGCGGAGAGTCACAGATGTAGC				
*TLR4*	F-GTGCCACATCCATACAATAGAAGA	57	217	JQ280465	93
	R-ATGGCCCAGATTCAGCTCCT				
**Reference Gene**					
*β-actin*	F-GAAATCCCTCCTCGCCAATCTGA	58	273	JN639846	98
	R-TGAAACGGAACAACACTGCCATCT				

^1^ IGF-I = Insulin-like growth factor-I, IL-6 = Interleukin-6, IL-10 = Interleukin-10, IL-12 = Interleukin-12, TNF-α = Tumour necrosis factor-alpha, TLR2 = Toll like receptor-2, TLR4 = Toll like receptor-4; * efficiency was calculated by the slope of the standard curve by the equation: E = 10^(−1/slope)^.

The relative fold expression of a target gene was computed, based on its real-time PCR efficiencies (E) or a static efficiency of 2, and the cycle threshold (*C*_t_) difference (∆) of mean control *versus* each unknown sample (∆*C*_t_ control − treatment) as described below [62] using β-actin (ACTB) as the reference housekeeping gene.

(1) $$\text{Fold Expression}=\;\frac{{\left(\text{E target}\right)}^{∆C\text{t target }\left(\text{control }-\text{ treatment}\right)}}{{\left(\text{E ref}\right)}^{∆C\text{t ref }\left(\text{control }-\text{ treatment}\right)}}$$

### 4.7. Statistical Analysis

Moisture loss, embryo organ and body weight, O_2_ consumption and HE data were analysed by one-way (for the treatment effect) and two-way analysis of variance (ANOVA), taking nutrients (NanoAg, Thr and Cys) and non-NanoAg as factors. The mRNA expression level (fold expression) of growth- and immune-related genes was analysed by one-way ANOVA. The analyses were carried out using the SPSS software package version 16.0. Differences in mean values were considered significant at the level of 95% (*p* < 0.05) and 99% (*p* < 0.01).

## 5. Conclusions

It can be concluded that NanoAg can act as a metabolic enhancer, but not as a growth promoter. NanoAg alone or in combination with amino acids administered *in ovo* improved the immune status of embryos, and thus can act as a potential agent for the enhancement of innate and adaptive immunity in chickens.
